# Membrane‐Associated Biomolecules for Synthetic Cell Signalling

**DOI:** 10.1002/cbic.70450

**Published:** 2026-07-08

**Authors:** Chelsea Dack, Bingkun Li, Charlie Newell, Michael J. Booth

**Affiliations:** ^1^ Department of Chemistry University College London London UK

**Keywords:** artificial cell, biomolecule, biosensor, cell signalling, lipid bilayer, membrane, nucleic acid, synthetic biology, vesicle

## Abstract

Membrane‐associated biomolecules, primarily proteins, are key enablers of communication, responsiveness, and complexity in natural living cells. Aiming to mimic these capabilities, there is growing interest in equipping bottom‐up synthetic cells with membrane‐associated biomolecular components. In this review, we focus on how proteins and nucleic acids have been associated with synthetic cell membranes, particularly lipid vesicles, to enable the transmission of signals across the membrane. We discuss strategies for anchoring these biomolecules into lipid bilayers and review how they can enable essential signalling mechanisms in synthetic cells, including cell tethering, the generation and fusion of vesicles, and signal transmission and transduction. We highlight how proteins offer native biological functionality, while nucleic acids may bring more modularity and control. Advancing this area will be essential for realising synthetic systems capable of studying natural communication mechanisms and unlocking applications in biosensing, therapeutics, and synthetic tissue engineering.

## Graphical abstract

Membrane‐associated proteins and nucleic acids are transforming synthetic cell communication, unlocking applications in biosensing, therapeutics, and tissue engineering. This review explores how these biomolecules enable complementary strategies for cell tethering, vesicle generation and fusion, signal transmission, and signal transduction. It highlights emerging hybrid approaches and key challenges towards increasingly sophisticated synthetic cellular behaviours.

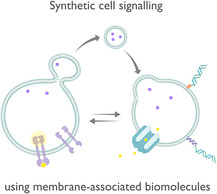

## 1 Introduction

**FIGURE 1:**
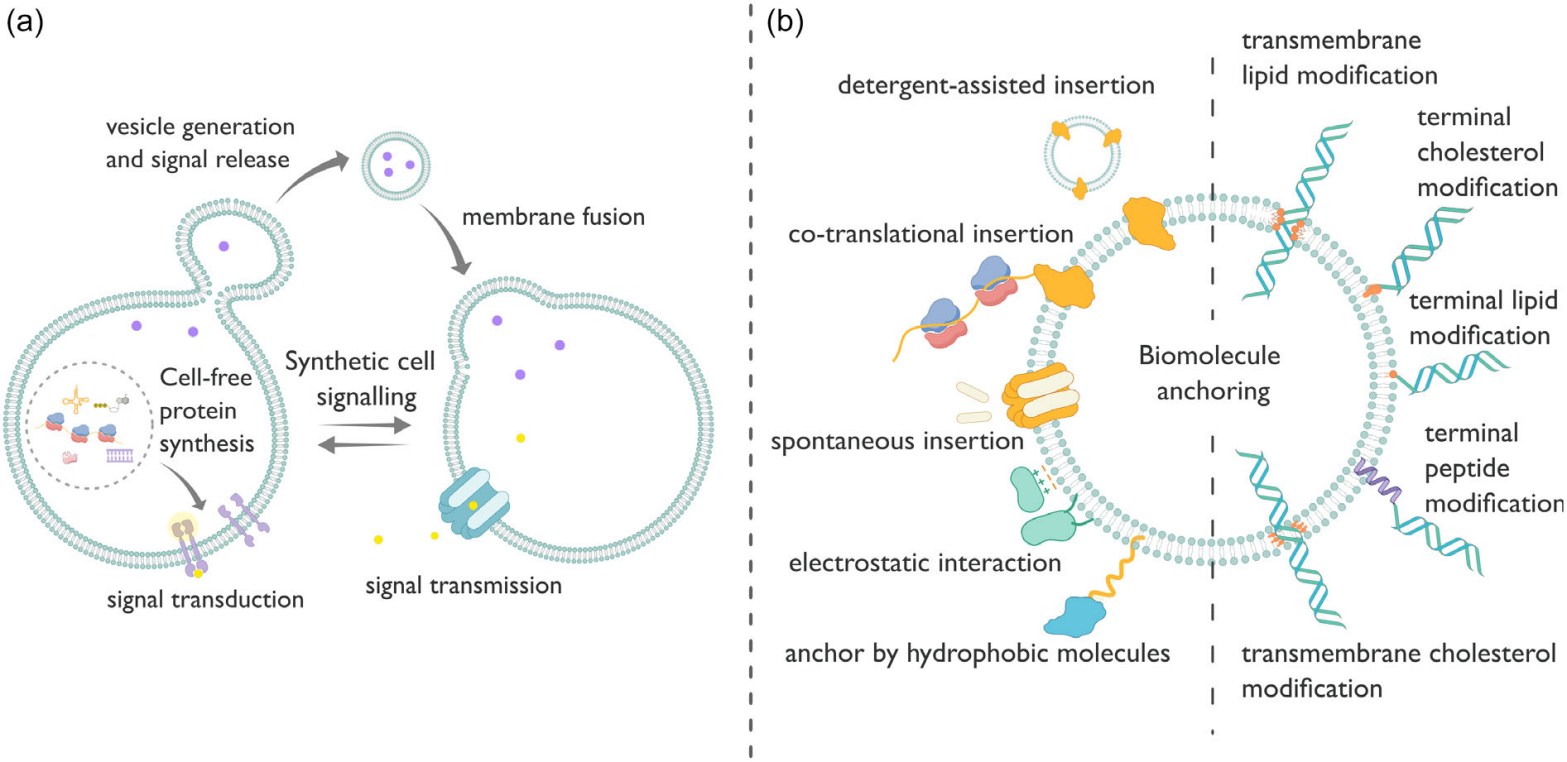
Membrane‐associated biomolecules enable key natural signalling mechanisms in synthetic cells. (a) Using either purified biomolecules or in situ expressed proteins, synthetic cells have been engineered to transmit, transduce, and shuttle information across membranes to drive signalling. (b) Diverse anchoring strategies exist to associate both protein and DNA to synthetic cell lipid membranes.

Bottom‐up synthetic cells are biomimetic compartments designed to reproduce selected features of living systems. Although membrane‐less variants exist, such as coacervates [1] and hydrogels [2], most are membrane‐bound. Membrane architectures include polymersomes [3], proteinosomes [4], and most commonly small, large, or giant unilamellar lipid vesicles (SUVs/LUVs/GUVs, 20 nm to 100 µm), which closely resemble natural membranes. By reconstituting native cellular machinery, repurposing biomolecules, or designing synthetic analogues, synthetic cells can mimic cellular behaviours such as communication (Figure 1a) [5]. Their modularity makes them powerful minimal systems for modelling biological processes [6] and engineering smart biological devices [7, 8, 9, 10].

In natural cells, the membrane is a dynamic signalling interface. Transmembrane signalling, via molecular transport or signal transduction [11], is essential for environmental responsiveness, cell–cell communication, and increasing system complexity [5, 12]. If synthetic cells are to achieve similar functionalities that are necessary for their application, transmembrane signalling is vital, yet it remains a major challenge. While passive diffusion can mediate signalling, such as with bacteria [8, 10], most biological signalling molecules are unable to passively permeate the lipid bilayer. In natural cells, membrane‐associated biomolecules play a central role in transmembrane signalling. They can directly interact with the signalling molecule for its transduction [13, 14], enable its direct passage [15, 16], or produce signal‐carrying vesicles for its transport [17, 18]. Functionalisation of synthetic cell membranes with such biomolecules is therefore essential for constructing these signalling pathways (Figure 1a).

Membrane‐associated proteins are the primary mediators of transmembrane signalling in nature. While synthetic amphiphiles, engineered polymers, and magnetic nanoparticles have been used to engineer signalling processes in synthetic cells [19, 20, 21], biomolecules have distinct advantages, such as their biocompatibility, ability to directly interface with cellular machinery and support genetic encoding, and their highly specific signalling functions. Membrane‐associated proteins can be incorporated into synthetic cells either through reconstitution of purified components or through an external or encapsulated cell‐free protein synthesis (CFPS) system (Figure 1). Derived from crude cell lysates or recombinantly purified elements [22], CFPS systems enable proteins to be synthesised from DNA templates. Excitingly, de novo protein design is further expanding this repertoire [23]. In parallel, nucleic acids—particularly DNA—have emerged as powerful alternatives. When modified with hydrophobic anchors, DNA strands can insert into lipid bilayers (Figure 1b), offering programmability, design flexibility, and diverse control mechanisms [24]. Membrane‐associated proteins and nucleic acids form a rapidly expanding toolkit for constructing signalling pathways in synthetic cells, which is critical for unlocking their applications as biological models and smart devices.

This review examines how membrane‐associated proteins and DNA have been used to engineer signalling in lipid vesicle‐based synthetic cells. It first describes strategies for anchoring these biomolecules within lipid bilayers (Figure 1b), then discusses their application in reconstructing cell tethering and fusion, vesicle generation, and transmembrane signal transmission (relay of a signal) and transduction (conversion of a signal from one form to another).

## 2 Membrane Anchoring

### 2.1 Proteins

Membrane‐associated proteins are either integral or peripheral (Figure 1b) [25]. Peripheral membrane proteins are water‐soluble and reversibly associate with the membrane [26]. Integral membrane proteins (IMPs) are inherently amphipathic, meaning their stability is dependent upon insertion into a lipid‐bilayer. The function of IMPs is often dictated by their orientation, and they regularly rely on the lateral pressure provided by the membrane to maintain a specific conformation. This makes them challenging to purify, as once they are removed from the membrane, they often denature or form non‐functional aggregates [27]. Consequently, synthetic biologists are limited to a select group of robust IMPs to reconstitute in synthetic cells [28].

Reconstituting IMPs into SUVs and LUVs is mostly done by the detergent‐assisted method. Purified IMPs are solubilised in detergent and then mixed with detergent‐stabilised lipid vesicles. The detergent is gradually removed, forcing the proteins to insert into the lipid bilayer to form proteoliposomes [29, 30]. These are often used as precursors to reconstitute IMPs into GUVs using the dehydration‐rehydration method. The proteoliposomes are dried onto a surface along with a mixture of lipids, forming a thin film. Upon hydration with an aqueous solution, the film swells into GUVs [31]. This process was later adapted for methods such as electroformation [32] and gel‐assisted swelling [33] to obtain higher yields. Several alternative strategies have been developed to avoid the dehydration step, which often denatures proteins prior to GUV formation. Direct detergent‐mediated incorporation involves adding detergent‐solubilised proteins directly to pre‐formed GUVs [34]. Fusion‐mediated insertion involves the spontaneous mergence of proteoliposomes with GUVs [35], driven by charge interactions [36, 37] or fusogenic agents [38]. This approach of reconstituting purified MPs into lipid compartments poses some persistent issues. For instance, lipid compositions affect activity of membrane proteins [27, 39, 40], the orientations of insertion are often not unidirectional [29, 41], and additional efforts are usually needed to ensure optimal insertion orientation [42].

Although purified IMPs are commonly reconstituted into synthetic cells, the majority are co‐translationally inserted in nature [43]. This is done by translocase and insertase systems, which guide the proteins into the membrane while preventing their aggregation [43, 44, 45, 46]. CFPS systems enable the reconstitution of these pathways in synthetic cells. SecYEG is a well‐characterised translocon present in *E.coli*. This has been expressed in synthetic cells to increase the insertion rate and function of IMPs [47, 48]. Co‐translational insertion can also be improved by engineering mRNA transcripts that bind cholesterol‐modified single‐stranded DNA embedded in the membrane (see below Section 2.2) [49]. Similarly, the process can be improved by artificially tethering ribosomes to the bilayer [50]. A minority of IMPs do not require co‐translational insertion and instead spontaneously insert into the membrane. These include the KcsA potassium channel [42] and a group of bacterial pore‐forming proteins, such as α‐hemolysin [51].

Peripheral membrane proteins (PMPs) are water‐soluble, so they can be directly reconstituted in synthetic cells, either from purified protein or a CFPS system. To associate with membranes, PMPs rely on either electrostatic interactions, lipid‐binding domains, protein–protein interactions, or an amphipathic helix that is protected from the cytoplasm [52]. Their function often requires specific lipid compositions and the presence of adaptor proteins [53]. Alternatively, non‐membrane proteins can be tagged with hydrophobic anchors that embed into synthetic cell membranes. Anchors include cholesterol, or lipids and fatty‐acids that mimic natural post‐translational modifications [54, 55]. Attachment chemistries can also be used, such as modifying lipids with nickel ligands to interact with protein histidine tags [56] or using lipids that can covalently bond with proteins [57, 58].

### 2.2 DNA

Unlike membrane proteins that can naturally insert into lipid bilayers, direct DNA‐membrane interactions typically require chemical functionalisation (Figure 1b). In the absence of cationic lipids or high concentrations of divalent cations that promote electrostatic attraction, the negatively charged, hydrophilic DNA backbone is repelled by hydrophobic lipids [59, 60]. This reliance on chemical modification limits dynamic membrane insertion within synthetic cells. Nevertheless, carefully designed nucleic acids can, in some cases, interact directly with lipid membranes without chemical modification [61]; further exploration of this ability suggests opportunities to expand the role of nucleic acids in synthetic cell systems. Indirect membrane associations can also occur, where protein‐conjugated [62] or aptamer DNAs bind to membrane‐anchored molecules such as protein receptors [63, 64, 65].

The chemical versatility of DNA enables a wide range of direct membrane anchoring strategies (Figure 1b). A primary method is through covalent conjugation with functionalised lipids. Maleimide‐thiol coupling is commonly employed and offers higher efficiency than traditional disulphide exchange between thiol‐modified lipids and DNA [59]. Copper‐free click chemistry has also been widely used, for example through conjugation of azide‐functionalised lipids with dibenzocyclooctyl‐modified DNA [66]. Beyond lipid conjugation, hydrophobic or amphiphilic DNA modifications can promote membrane association. These include hydrophobic peptides [67], polymers [68], long lipid chains [69], and small hydrophobic groups, such as cholesterol, porphyrins, or ethylated phosphorothiorate modifications [59, 69, 70]. These anchors allow control over DNA insertion depth and stability [68]. The DNA anchor can also reversibly direct localisation to specific lipid domains within phase‐separated membranes [71, 72, 73].

The choice of DNA anchoring strategy depends strongly on the intended application and must balance stability, orientation, and ease of synthesis. Cholesterol remains the most widely used anchor due to its rapid insertion kinetics, commercial availability, and design flexibility [59, 69, 70]. Although it forms weak membrane interactions [66], anchoring strength can be increased by incorporating multiple cholesterol moieties [74]. Solubility is improved using triethylene glycol spacers [75], and aggregation is minimised through careful sequence design [76]. Cholesterol positioning further enables control over DNA orientation, ranging from surface tethering to membrane spanning configurations [70]. However, cholesterol‐DNA insertion can perturb membrane stability [75], and high loading densities may alter DNA conformation [77].

### 2.3 Summary

Diverse protein and DNA membrane‐anchoring strategies have established a solid foundation for engineering signalling mechanisms in synthetic cells (Figure 1b). As seen with proteins and CFPS, future exploration of inherently membrane‐interacting nucleic acids [61] and in situ RNA synthesis [78, 79] will expand this toolbox further. Combined with wider advances, such as magnetic control over gene expression in synthetic cells [21], new avenues for spatiotemporal control and integration with up‐ and downstream signalling mechanisms are emerging for both biomolecules. However, the inefficient reconstitution of more complex membrane proteins is a major limitation. For DNA, minimising its susceptibility to environmental degradation by nucleases, through use of chemical modifications used in nucleic acid therapeutics [80], will be critical for long‐term and in vivo applications.

## 3 Cell Tethering and Vesicle Fusion

**FIGURE 2:**
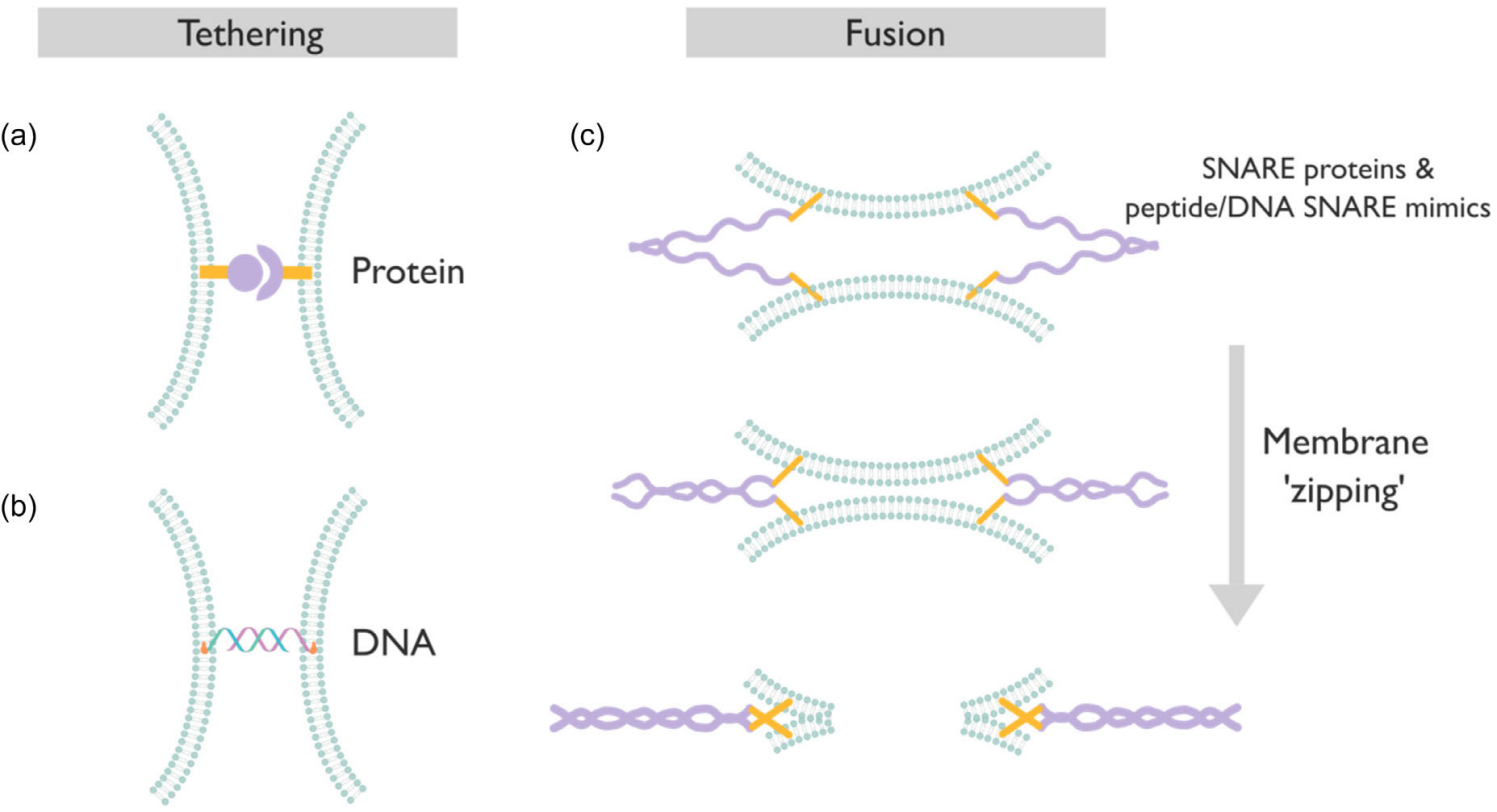
Tethering and fusion of synthetic cells. (a) Membrane‐anchored proteins can tether cells through the formation of non‐covalent and covalent linkages. (b) Nucleic acids anchored to the membranes of synthetic cells can initiate cell–cell tethering through complementary Watson‐Crick‐Franklin base pairing. (c) Natural SNARE proteins, along with synthetic peptides and DNA mimics, have been reconstituted into synthetic cells to mediate the fusion of opposing membranes.

In nature, membrane‐associated biomolecules facilitate tethering and fusion between neighbouring cells, and between cells and vesicles. Tethering is a prerequisite to membrane fusion, and it also maintains tissue integrity [81] and enables contact‐dependent signalling [82]. Fusion instead enables bulk signal delivery and cytosolic exchange through membrane mergence [83]. Replicating these capabilities in synthetic cells (Figure 2) enables multicellular architectures and behaviours, including signalling circuits reliant on physical contact and programmed spatial organisation.

### 3.1 Protein‐Mediated Membrane Tethering

Natural cell tethering is primarily done using cell adhesion molecules (CAMs). These are transmembrane proteins that interact to bind cells together and, in some cases, also initiate signalling [84]. CAMs are divided into two main families: the integrins and cadherins. Integrins specifically mediate cell attachment to the extracellular matrix [85]. They have been reconstituted into GUVs as a model system to study the dynamics of integrin‐mediated adhesion [86, 87] or the self‐assembly of adhesion complexes [88]. Cadherins instead mediate attachment between cells [89]. The extracellular domains of E‐cadherin have been integrated into GUVs and supported lipid bilayers to probe cis and trans interactions in cell–cell adhesion (Figure 2a) [90]. Recent work has begun to use these molecules to also facilitate intercellular communication: ectodomains of E‐cadherin were used to promote the docking of connexin channels, enabling the movement of signalling molecules between synthetic cells [91]. This may represent a new avenue for reconstituting natural communication pathways in synthetic cells.

Natural cells also possess soluble tethering factors. These are different to CAMs, as they primarily act to connect lipid bilayers rather than forming stable tissues. Atg23 is a natural PMP that acts as a tethering factor to form the autophagosome: it was reconstituted in GUVs where it successfully tethered Atg9‐containing SUVs [92]. LecA is a bacterial protein that participates in host‐cell interactions and biofilm formation: it was reconstituted in GUVs to form synthetic cell tissues built from lecA‐mediated intercellular junctions [93]. Synthetic protein‐ligand and protein–protein interactions can also control cell tethering. One of the earliest examples of vesicle tethering was achieved in SUVs using the strong non‐covalent linkage between biotin‐streptavidin [94]. Non‐covalent cell tethering can also be carried out using the iLID‐Nano protein pair, which bind upon irradiation with blue light: iLID and Nano were anchored to the membranes of separate GUV populations and tethered upon irradiation [95]. Tethering can also be engineered through the formation of a covalent linkage. The SpyTag‐SpyCatcher pair form a spontaneous isopeptide bond when in proximity: SpyTag and SpyCatcher have been modified to insert into the bilayers of two different GUV populations, causing GUV tethering upon mixing [96].

### 3.2 DNA‐Mediated Membrane Tethering

DNA‐mediated vesicle–vesicle tethering was first demonstrated in lipid vesicles using complementary membrane‐anchored DNA strands, whose hybridisation brought opposing vesicles into close contact (Figure 2b) [97]. The tuneable DNA tethers allow precise control of intermembrane distances, aiding protein‐mediated fusion between synthetic cells [98]. This tethering approach has been demonstrated with living cells, where they can create defined clusters [99] and three‐dimensional microtissues [100] to promote and study intercellular signalling [99, 101]. Adjustable DNA linkers can also associate with membrane‐anchored proteins to study living cell binding strength [62].

A further advantage of DNA‐mediated tethering is its dynamic reversibility, with hybridisation intrinsically controlled thermally or by ionic strength [97]. Strand displacement reactions provide a powerful strategy to fine‐tune tethering kinetics [102] or to reversibly disrupt aggregates [103, 104], including in response to microRNAs for biosensing applications [105, 106]. Alternatively, chain hybridisation reactions can programmatically regulate DNA tether assembly [104] and photoresponsive polymer membrane anchors can enable light‐induced dissociation [68]. In living cells, use of catalytic DNAzymes in the DNA tethers demonstrated responsiveness to specific metal cofactors [107]. However, despite their modularity and predictability, DNA‐based tethers cannot fully recapitulate the complexity of natural protein‐mediated adhesion systems.

### 3.3 Protein‐Mediated Membrane Fusion

Vesicle‐membrane fusion is vital for cellular communication. By enabling the transport of molecules between cells and compartments, key processes such as intracellular trafficking and endo/ exocytosis exist [108]. Eukaryotic membranes do not spontaneously fuse and instead rely on soluble *N*‐ethylmaleimide‐sensitive factor attachment receptor (SNARE) proteins to zip the membrane leaflets together (Figure 2c) [83]. Neurotransmission is a primary example of this process: SNARE proteins assemble across synaptic vesicle and cell membranes, fusing them together, resulting in the extracellular release of a neurotransmitter [109]. Researchers have reconstituted native SNARE proteins into synthetic cells to drive vesicle fusion in non‐living systems [110, 111]. These studies resemble the in vivo formation of the SNARE complex by integrating vesicle‐SNARE proteins into SUVs, and cell membrane‐SNARE proteins into GUVs [112]. Fusion kinetics have been achieved close to that of the rapid natural neuronal exocytosis [113].

SNARE mimics have been engineered that are easier to reconstitute and are required at a lower density in the membrane (Figure 2c). These are referred to as peptides ‘K’ (KIAALKE) and ‘E’ (EIAALEK) and are anchored to membranes by engineered hydrophobic domains [114]. Fusion mediated by zipper‐like coiling was initially demonstrated in SUVs, and more recently in LUVs and GUVs [115]. Fusion rate is improved by increasing the peptide length [116] or by changing the anchor domain [117, 118]. Fusion efficiency can be improved by bridging opposing membranes using DNA tethers (described in Section 3.2) [98]. Controlling fusion typically requires additional mechanisms, such as proteolytic enzyme cleavage [119], steric blocking with a light‐sensitive lipid conjugate [120], or pH‐responsive peptides [121]. More recently, a study engineered a simpler method by designing orthogonal peptide pairs [122]. SNARE mimics have been applied to study membrane fusion processes [118, 123, 124], to deliver nucleic acid therapeutics and proteins to living cells [125, 126], and to fuse vesicle populations to initiate genetic cascades [127]. Proteolytic enzyme biosensors have also been developed [119], as well as synthetic beta cells that release insulin in response to a hyperglycaemic environment [128].

### 3.4 DNA‐Mediated Membrane Fusion

When hybridising in a zipper‐like fashion, DNA tethers can drive membrane fusion (Figure 2c), mimicking the function of SNARE proteins [104, 129, 130, 131, 132, 133]. DNA‐mediated vesicle fusion can deliver exogenous proteins to mammalian cells in vitro [104, 134], programmatically load extracellular vesicles for therapeutic delivery [135], and sequence‐orthogonal DNA pairs can selectively fuse vesicle populations [136, 137]. Modifying lipid composition [138] or temperature can enhance fusion, as can increasing the surface density of membrane‐anchored DNA [132], which can be promoted using lipid phase separation [139]. Membrane anchor strength is critical for generating sufficient mechanical force and depends on the number and type of hydrophobic anchors attached to each DNA strand [130, 140]. Efficiency can also depend on DNA sequence [69] and be improved by double‐stranded DNA ‘tendrils’ that prevent strand collapse onto the bilayer surface [138].

The DNA strands can be sterically shielded by proteolytically cleavable polyethylene glycol chains, enabling small molecule‐activated fusion [141]. Photocleavable modifications, such as *o*‐nitrobenzyl groups, allow activation of DNA hairpins using ultraviolet or near‐infrared light when combined with upconversion nanoparticles [134]. More complex control schemes integrate photocaged hairpins with pH‐responsive triplex‐forming DNA motifs to achieve dual stimuli‐responsiveness [142]. Strand displacement can also enable precise temporal fusion control by triggering the removal of blocking DNA strands [138].

### 3.5 Summary

Tethering has provided an effective strategy to promote contact‐dependent mechanisms in synthetic cells, such as connexin pore formation [91] and fusion [98]. It has also demonstrated potential for the creation of defined synthetic tissues [100]. Synthetic cell fusion has equally emerged as a powerful tool, for studying SNARE‐mediated signalling and membrane dynamics [118, 123], developing novel biosensors [105, 106, 119], and enabling therapeutic delivery [104, 125, 128, 134]. DNA‐based mechanisms provide greater programmability, spatiotemporal control strategies, and dynamic reversibility. However, proteins possess superior complexity, allowing integration with more sophisticated signalling cascades. Hybrid approaches may provide a promising route forwards, harnessing DNA's design flexibility with protein's native functional sophistication [62].

## 4 Membrane Sculpting for Vesicle Generation

**FIGURE 3:**
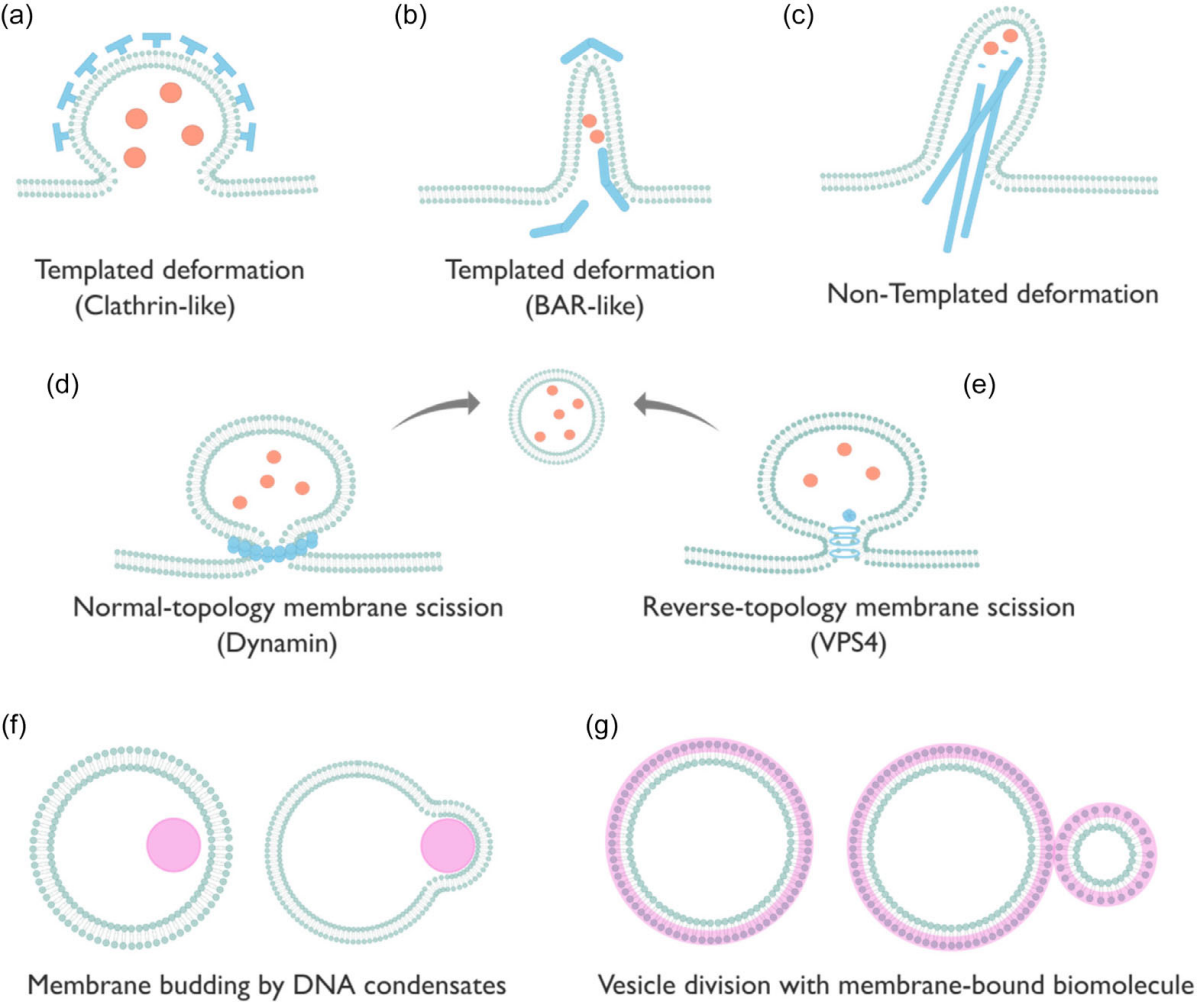
Mechanisms of synthetic cell membrane sculpting for vesicle generation. Templated membrane deformation induced by (a) Clatherin‐like, and cytoskeleton‐like structures. (b) BAR‐like structures. (c) Non‐templated membrane deformation induced by cytoskeleton‐like structures. Vesicle scission induced by (d) dynamin or (e) Vps4. (f) Budding induced by phase separation. (g) Scission induced by unspecific membrane adsorption.

Most fusion‐mediated communication requires vesicles. As with vesicle fusion, vesicle budding requires energy to overcome tension and to deform the membrane. Vesicles can either bud from a planar membrane or a curved membrane following tubulation [18]. In nature, these processes are controlled by membrane‐sculpting proteins [52]. These are primarily PMPs that sculpt the membrane through two major mechanisms: templated deformation, which is driven by proteins assembling into rigid scaffolds [143, 144], and non‐templated deformation, driven either by physical forces (such as pushing and pulling) [145, 146] or passive forces (such as pressure caused by crowding) [147]. The vesicle is then typically pinched off by scission machinery [148]. Reconstituting vesicle generation in synthetic cells (Figure 3) enables bulk, long‐distance signal exchange towards multi‐factor signalling behaviours, such as phenotype differentiation.

### 4.1 Protein‐Mediated Membrane Deformation

The final geometry of vesicles formed by templated deformation is determined by the structure of the protein scaffold. Clathrin and COPI/II are specialised for generating uniform spherical carriers involved in endocytosis or intracellular trafficking, respectively. Clathrin coats can generate spherical buds in LUVs [149], or GUVs provided there is a balance between membrane elasticity and polymerisation energy (Figure 3a) [53]. Similarly, minimal sets of ESCRT‐III proteins have generated intraluminal vesicles in GUVs by forming spiral scaffolds [150]. Alternatively, BAR‐domain proteins, such as IRSp53, sense and stabilise membrane curvature, forming tubules rather than spheres (Figure 3b) [151, 152].

Non‐templated deformation is either performed actively, by non‐specific mechanical actuators [145, 146], or passively, through steric pressure [147]. Both processes produce variable structures such as protrusions, blebs, and nanotubes. Mechanical actuators include eukaryotic cytoskeletal proteins such, as actin, microtubules, and actomyosin, and their bacterial homologs, MreB, FtsZ, and BtubA/B [145, 146]. They function by either protruding the membrane outwards using polymerisation force, or blebbing the membrane inwards using contractile force. For example, inwards constriction has been driven by reconstituting contractile rings, from either pre‐purified eukaryotic actomyosin or through the CFPS of bacterial FtsZ [153, 154]. Conversely, outwards tubulation has been induced in GUVs through the CFPS of BtubA/B (Figure 3c) [155]. GUVs have also been deformed into oval and rectangular shapes through a combination of the internal expression of MreB, and molecular crowding by pegylated lipids located in the membrane [156]. Furthermore, molecular crowding has been shown to cause GUV membrane deformation in the absence of a mechanical actuator. For example, when introduced to GUVs at low densities, large, intrinsically disordered proteins generated membrane curvature and vesicle budding (Figure 3g) [157].

The final step in vesicle formation is membrane scission. In nature, this is typically carried out by specialised enzymes, such as dynamin for cytoplasmic vesicles or Vps4 for extracellular vesicles. In vitro, dynamin has been shown to split dumbbell‐shaped vesicles (Figure 3d) [158], and Vps4 has catalysed the scission of ESCRT‐III nanotubes generated from GUVs (Figure 3e) [159]. In addition, proteins that form contractile rings during cell division, such as actomyosin [160], might be repurposed for vesicle scissoring. Scission can also occur passively by lipid phase separation (Figure 3f) [161], or friction‐driven pearling instabilities [162].

### 4.2 DNA‐Mediated Membrane Deformation

As with membrane‐anchored proteins [157], GUVs can undergo deformation upon the insertion of membrane‐anchored DNA (Figure 3g) [163]. Furthermore, owing to the structural versatility of DNA, a broad range of complex and dynamic membrane deformations can be achieved, relying on pre‐formed nanocages as templates for vesicle assembly [164] or protein mimics that shape the membrane post‐synthesis, often exceeding the diversity accessible with proteins [60]. Membrane‐anchored DNA nanostructures have successfully mimicked several classes of membrane‐deforming proteins in GUVs, including BAR‐domain curvature inducers (Figure 3a) [165], dynamin‐ and ESCRT‐like tabulators and scission mimics (Figure 3d) [67], and clathrin‐inspired network architectures (Figure 3b) [166]. The degree of deformation is enhanced by DNA curvature, anchor strength, and surface density [24]. Dynamic control can be achieved by harnessing inherent DNA properties, via salt concentration [167], sequence design, and temperature [168], or by light‐responsive chemical modifications [169]. Self‐contracting DNA lattices on the vesicle surface can also control membrane shape [170]. Similarly, in situ RNA synthesis within GUVs has enabled the dynamic assembly of membrane‐anchored nucleic acid cytoskeleton‐like structures (Figure 3c) [78, 79].

However, complete vesicle formation mediated by DNA‐based protein mimics has not yet been unequivocally demonstrated. As an alternative strategy, DNA nanostructures that accumulate at phase‐separated lipid interfaces can produce vesicles, via strand displacement and osmotic pressure modulation [71]. Similarly, photoactivatable lipid‐anchored DNA condensates inside synthetic cells can trigger light‐controlled membrane budding (Figure 3f) [171].

### 4.3 Summary

Although strategies for achieving vesicle production are currently limited using either biomolecule, diverse membrane deformation mechanisms provide a strong foundation. Rapid advances in *de novo* protein design and the emergence of artificial protein cages are increasing the feasibility of protein‐mediated approaches, improving in vitro assembly and overcoming reliance on complex accessory factors [172]. Similarly, development of RNA‐based systems [78, 79], self‐contracting DNA arrays [173], dynamic DNA lattices [170], establish a conceptual framework for introducing dynamic control vital for efficient function. Integration of vesicle‐generating systems with programmable tethering and fusion modules could yield fully autonomous synthetic cells, capable of adaptive morphology, targeted cargo delivery, and coordinated communication—paralleling natural extracellular vesicle systems [174].

## 5 Transmembrane Transport: Pores, Channels, and Pumps

**FIGURE 4:**
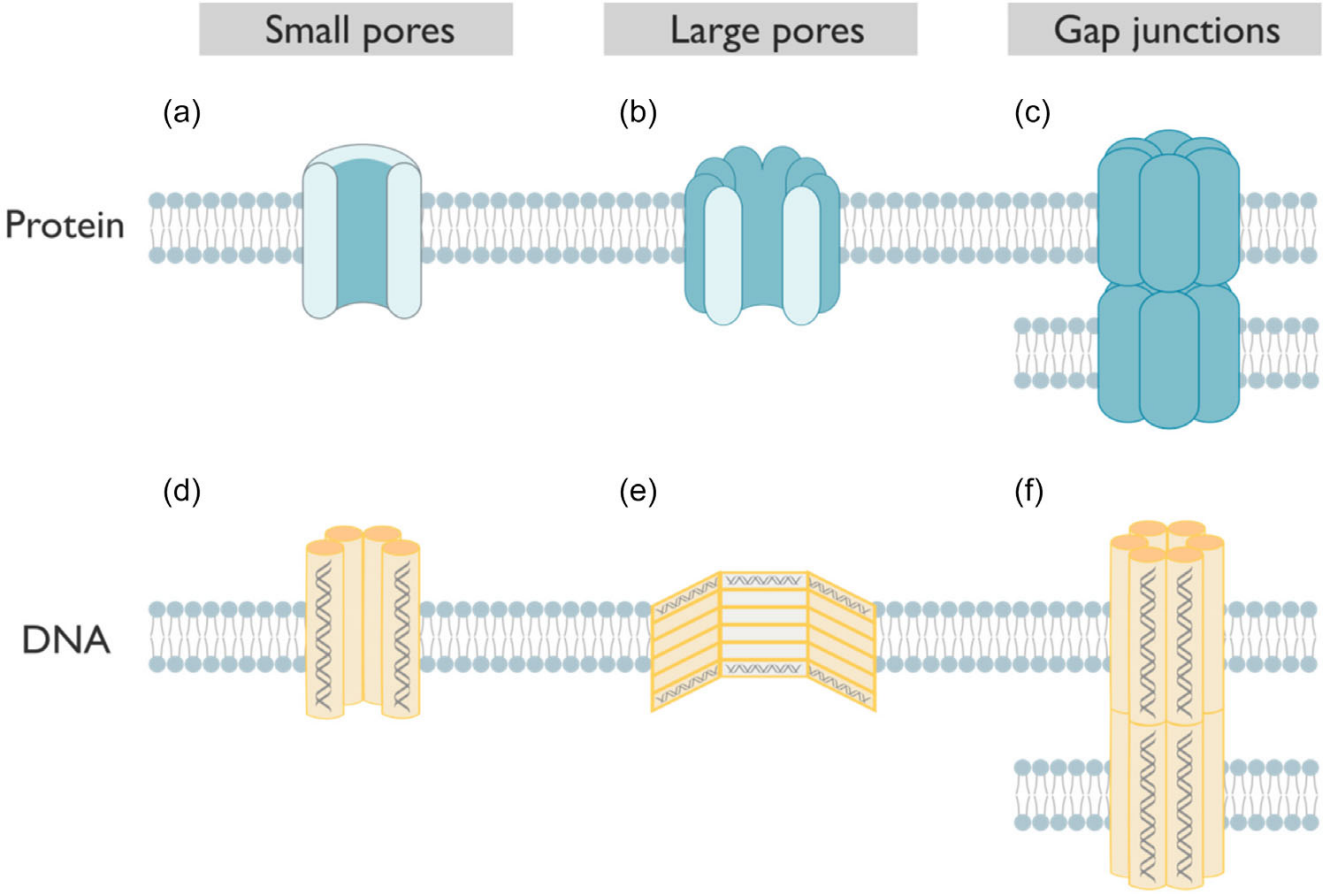
Varied sizes of protein and DNA pores, enable the direct transmission of molecules between synthetic cells, and with the extracellular environment. (a) Small protein pores, such as KvaP, α‐hemolysin, and GLUT1/2, enable the selective or non‐selective transport of ions or small molecules. (b) Large protein pores, including Perfringolysin O, enable the non‐selective transport of ions, small molecules, and proteins. (c) Gap junctions, for example connexins, and engineered α‐hemolysin bridge opposing membranes enabling the transport of ions and small molecules between connected cells. (d) Small DNA pores, such as six‐helix bundles, enable voltage‐gated transport of ions. (e) Large DNA pores including DNA origami cubes or transmembrane microchannels, enable the non‐selective transport of ions, small molecules, and proteins. (f) Similarly to protein gap junctions, dimerisation of DNA pores can bridge opposing membranes permitting the exchange of intracellular cargo.

Transmembrane signalling can occur via direct transmission, which involves the movement of ions and small molecules across the lipid bilayer. This is facilitated by a superfamily of proteins including channels, transporters, and pore‐forming proteins (PFPs) (Figure 4a,b). In neuronal cells, the movement of ions through voltage‐gated channels generates action potentials that carry information [175], and in the immune system, the transport of calcium ions through specific channels activates T‐cells [176, 177]. In synthetic cells, transmembrane transmission enables fast, reversible transport and directly couples internal biochemical processes to the external environment (Figure 4).

### 5.1 Protein Transmembrane Transport

#### 5.1.1 Passive Protein Pores and Transient Peptide Permeabilisers

PFPs are a broad class of molecules that participate in pathogenesis, host response and immunity, and programmed cell death [178, 179, 180]. Upon binding to a membrane, PFPs transition from soluble monomers into transmembrane pores, causing the influx and efflux of ions and molecules. Pore‐forming toxins produced by many pathogenic bacteria are a well‐characterised class of PFP that are often reconstituted in synthetic cells. α‐hemolysin, derived from *Staphylococcus aureus*, assembles into heptamers forming nanopores of 1.4 nm in diameter. These allow the diffusion of molecules smaller than 2 kDa [51]. α‐hemolysin serves as a gold standard for verifying vesicle unilamellarity [181, 182, 183] and is used extensively to facilitate signalling. It has transported molecules between separate vesicles [95, 184, 185], shuttled internal molecules between compartments [186, 187, 188, 189], and received environmental cues that activate internal chemical reactions [95, 190]. Perfringolysin O (PFO) is another commonly used pore that is a member of the cholesterol‐dependent cytolysin family. PFO nanopores are some of the largest known, with diameters of 25–30 nm [191]. CFPS of PFO was used to release a large neurotrophic factor protein, BDNF, from synthetic cells to trigger neuronal differentiation in neighbouring living cells [9].

Membrane‐active peptides also facilitate direct transmission. These are amphipathic and less than 50 amino acids in size. They either disrupt, reside in, or pass through membranes [192, 193]. Two prominent classes of membrane‐active peptides are antimicrobial peptides and cell‐penetrating peptides. Mellitin is a well‐studied antimicrobial peptide isolated from honeybee venom [194]. It has been used to permeabilise synthetic cell membranes for the release and uptake of cargo [195, 196]. At micromolar concentrations, it creates stable pores that transport molecules up to 10 kDa, and at nanomolar concentrations, it creates transient pores that can only transport ions [197]. Cell‐penetrating peptides can instead cross membranes without disrupting them. Their translocation is often mediated by the formation of a transient pore, but the exact mechanisms are being actively researched [198, 199]. They have been shown to translocate a variety of cargoes across GUV membranes to activate signalling [200, 201, 202].

#### 5.1.2 Gated and Stimuli‐Responsive Protein Pores

In nature, certain pores can be formed or activated in response to environmental cues. These are introduced into synthetic cells for functional assays or to engineer signalling pathways consisting of orthogonal sensors. Purified KvAP, a voltage‐gated potassium channel, has been successfully reconstituted into GUVs, which open and close in response to a change in membrane potential [203]. The bacterial mechanosensitive channel MscL forms a large and transient pore in response to increased membrane tension [204, 205]. GUVs have been prepared containing a phospholipase enzyme, and MscL‐functionalised SUVs encapsulating a dye [206]. Upon activation of the phospholipase, membrane tension of the SUVs was altered, which activated the channel and released the dye. MscL has also been used to activate CFPS in GUVs, by triggering the influx of an inducer molecule in response to a change in osmotic pressure [207]. Various native proteins have also been engineered to be stimuli‐responsive, although this has predominantly been demonstrated in living cells where reconstitution is easier [208].

#### 5.1.3 Transporters and Pumps

Transporters recognise specific substrates and are either passive or active. In nature, they have several roles, including nutrient scavenging [209], toxin removal [210], and energy generation [211]. Some of these functionalities have been reconstituted in synthetic cells. The passive glucose transporters GLUT1/2 have been reconstituted into GUVs to uptake glucose molecules [128, 212]. Passive transporters can also exchange substrates. LUVs were created containing a mitochondrial ADP/ATP carrier protein [213]. This facilitated nucleotide exchange, establishing an ATP‐cross‐feeding reaction between two vesicle populations. Active transporters include mitochondrial Complex I and ATP synthase, which cooperate to produce ATP. These were co‐reconstituted in LUVs to generate energy by cellular respiration [214].

#### 5.1.4 Inter‐Cellular Junctions

Gap junctions are channels found in animal cells that span two adjacent cell membranes [215]. The connexins are a well‐characterised family that form upon the contact of two hemichannels composed of six protomers (Cx) each (Figure 4c) [216]. In nature, adhesion molecules are necessary to bring the membranes into stable contact prior to forming the channel [217]; however, they are not necessary when vesicles containing hemichannels adhere to living cells [218]. Cx43 hemichannels have been reconstituted in GUVs using encapsulated CFPS systems to release an internal dye [219]. Complete connexins have also been reconstituted in GUVs. Two GUV populations were prepared that contained protease‐responsive Cx43 or Cx32 hemichannels [91]. Functional connexins were formed upon light‐activated release of the corresponding protease, which enabled formation of gap junctions between synthetic cells and the transfer of molecules. Gap junctions have also been engineered from proteins that do not natively span adjacent membranes. Using connexons as a model, α‐hemolysin pores were engineered to couple in a cap‐to‐cap orientation through spontaneous disulphide bond formation between two mutant cysteine residues [220]. The dimeric pore successfully bridged adjacent LUV membranes and planar lipid bilayers with LUVs to form conductive pathways.

### 5.2 DNA Nanopores

A diverse repertoire of DNA nanopores has been developed to mediate molecular transport across GUV membranes, ranging from ion channels (Figure 4d) [163, 221, 222, 223] to large protein‐accommodating pores (Figure 4e) [224, 225]. Design strategies vary considerably; in GUVs, ion‐transporting DNA nanopores can be archetypal six‐helix‐bundles [222], four DNA‐tile structures [163], or single‐stranded constructs that function via secondary structure formation [223] or toroidal membrane pores [221]. For larger cargo, DNA origami cubes [226] or transmembrane microchannels [227] have been embedded in GUV membranes. Self‐contracting DNA lattices on the vesicle surface can also trigger cargo release [170]. Moreover, DNA nanopore dimerisation can bridge adjacent lipid bilayers, permitting controlled content exchange between GUVs (Figure 4f) [228]. Mimicking gap junction‐like communication, such DNA systems are an important step towards interconnected synthetic cell networks.

DNA nanopore activity can be regulated in GUVs by a wide range of stimuli, including temperature [229], specific ions [223], and voltage [222]. Incorporating aptamer sequences or photoresponsive moieties, such as azobenzene, further extends control, allowing GUV pore activation by specific proteins [230] or light [231], respectively. Pore gating can also be achieved using blocking DNA strands [232] or invading DNA strands in solution [233] and on membranes [234], often through opening of a DNA ‘lid’. Alternatively, membrane insertion itself can be regulated by assembling pores from free subunits [235] or by exposing lipid flaps that facilitate anchoring [236].

Despite these advances and high versatility, most DNA nanopore demonstrations in GUVs remain as proof‐of‐concept and have yet to be applied for functional applications. Complex DNA pores are typically expensive, slow to assemble, and may require external stimuli to achieve complete membrane insertion [24, 237], such as detergents or electroporation [208, 237]. The hydrophobic modifications required for membrane insertion can also disrupt DNA self‐assembly or promote aggregation [76, 238]. Furthermore, achieving molecular selectivity beyond size exclusion or stimuli‐induced gating remains challenging [24], due to intrinsic ion permeability and environmental sensitivity [208, 239].

### 5.3 Hybrid DNA‐Protein Nanopores

Hybrid nanopores combine the more efficient membrane‐inserting properties of peptides with the structural precision and programmability of DNA [240, 241, 242]. By conjugating peptide pore monomers to a DNA scaffold, stable and monodisperse pores can assemble in situ within GUVs, forming channels of defined size [243]. Owing to the flexibility of DNA design, such hybrid structures allow fine‐tuning of pore dimensions and cargo selectivity, while also offering the potential for stimuli‐responsiveness and the integration of targeting motifs or other functional domains.

### 5.4 Summary

Direct signal transmission via protein units has enabled responsiveness to environmental cues [244], phenotype differentiation [9], and ATP energy regeneration [214] in synthetic cells, yet their efficient membrane insertion remains a significant barrier. Although DNA nanopores offer attractive design flexibility and stimuli‐responsiveness, these are yet to be applied for functional applications in synthetic cells. Future advances regarding both protein‐ and DNA‐based units depend on improved selectivity and membrane integration. A promising route forward may be to rely on DNA's programmability to assemble more complex protein pores from simpler membrane‐inserting protein units, as demonstrated in hybrid pore systems [240, 243].

## 6 Signal Transduction

**FIGURE 5:**
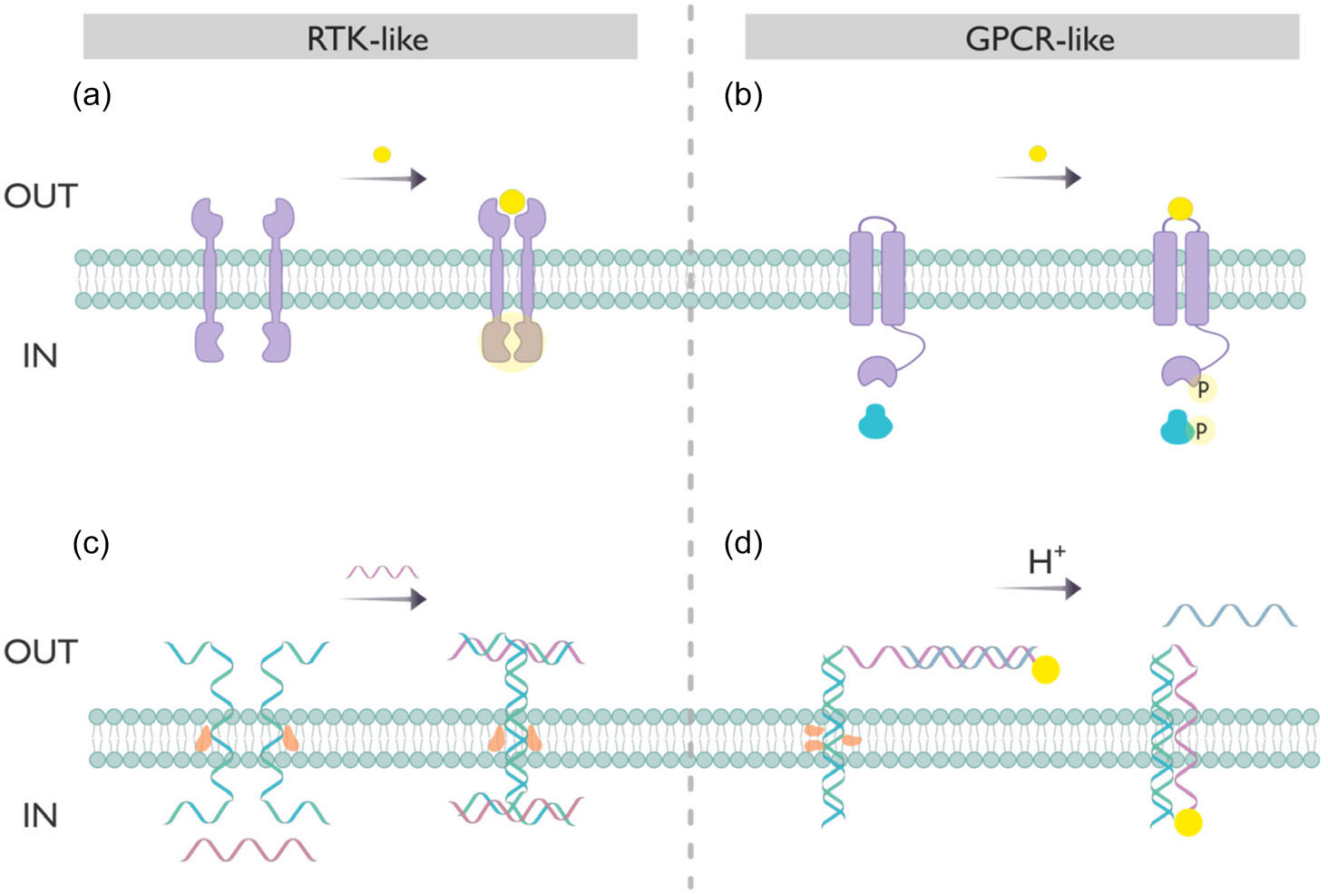
Mechanisms of signal transduction in synthetic cells. (a) Synthetic, RTK‐like protein receptors dimerise in the presence of an external ligand, producing an internal fluorescent response. (b) GPCR‐like receptors, as well as bacterial two‐component systems, activate downstream protein phosphorylation cascades upon recognition of an external ligand. Protein phosphorylation typically results in the internal regulation of gene expression. (c) DNA analogues of RTK receptors, dimerise upon binding to a complementary signal strand. Dimerisation reconstitutes split catalytic motifs to amplify an internal response. (d) Under acidic conditions, a DNA‐based GPCR undergoes conformational rearrangement to form a DNA triplex. This promotes the translocation of a signal strand from the external to the inner membrane leaflet.

In nature, most cell signalling occurs by signal transduction, rather than signal transmission, and is facilitated by transmembrane receptors [11]. While transmission is the relay of signal, transduction involves conversion of the signal from one form to another. This enables amplification and integration, meaning one signal can cause several outcomes or several signals can cause just one [245]. This involves the receptor activating intracellular proteins to propagate the signal [246]. The cell is then able to process information to make complex decisions [247], whilst its internal environment is protected from the stimulus. Implementing signal transduction in synthetic cells (Figure 5) opens routes to hierarchical and orthogonal control architectures while preserving compartment stability and integrity.

### 6.1 Protein‐Based Transmembrane Signalling Units

In eukaryotic cells, the major transmembrane receptors are G protein‐coupled receptors (GPCRs) and receptor tyrosine kinases **(**RTKs). GPCRs undergo a conformational change when bound to an extracellular ligand. This activates an intracellular G protein that generates secondary messengers to broadcast the signal [14]. RTKs instead dimerise and auto‐phosphorylate upon ligand binding. This initiates intracellular phosphorylation cascades to produce an outcome [13]. In synthetic cells, it is challenging to recreate these pathways because they are a complex mixture of proteins, and the native transmembrane receptors themselves are difficult to reconstitute from purified proteins or through CFPS. Instead, GUVs have been functionalised with protein constructs that resemble RTKs (Figure 5a). Two enzyme subunits were attached to a transmembrane domain and an antibody tag [248]. The enzyme was only activated once two constructs had dimerised to form a functional enzyme tetramer. This was induced by a bivalent antibody signal. The signal was transduced into the activation of the enzyme and the production of a fluorescent molecule. Alternatively, prokaryotes use two component systems, where a transmembrane receptor detects a signal then phosphorylates a response regulator that subsequently activates gene expression. CFPS was used to reconstitute the NarX‐L system in LUVs and GUVs (Figure 5b) [249]. A nitrate signal was transduced into the synthesis of nanoluciferase, which generated a fluorescent output. NarX was engineered to also sense iron and nickel.

### 6.2 DNA‐Based Transmembrane Signalling Units

DNA analogues of RTK proteins typically consist of single‐stranded DNAs anchored in GUV membranes via cholesterol moieties (Figure 5c) [250, 251] or lipophilic 12‐carbon spacers [252]. In response to external triggers—including invading DNA strands [251], small molecules [252], or ions [250]—these RTK‐like DNAs dimerise to activate split catalytic motifs within the vesicle. These catalytic domains, such as DNAzymes [251] or G‐quadruplexes [250, 252], can perform sequence‐specific cleavage or peroxidase‐like reactions, respectively, amplifying intracellular signals like fluorescence [251, 252] or cytoskeletal polymerisation [250]. Inherently, these small catalytic groups limit signal amplification compared with multi‐step enzymatic signalling pathways [253]. Although demonstrated in natural cells, advances have increased the functional sophistication of these RTK‐like DNA systems, by incorporating logic‐gate architectures for multi‐input control [254]. Nonetheless, their performance remains limited by two‐dimensional receptor diffusion and the variability in DNA orientation in the membrane [255].

A membrane‐anchored GPCR‐like DNA has been shown to translocate a third strand to the inner membrane leaflet to initiate signal amplification in a pH‐dependent manner (Figure 5d) [256]. While effective, this strategy requires multiple hydrophobic anchors and introduces additional structural complexity relative to simpler RTK‐like designs [253, 255]. A more minimal approach exploits the spontaneous translocation of cholesterol‐DNA across lipid bilayers [255]. Triggered by membrane defects and enhanced by glycerol, these DNAs migrate to the inner membrane leaflet where they can activate downstream processes such as RNA transcription [255]. Despite these advances, reproducing the specificity and complexity of native protein receptors remains a major challenge, particularly for sensing complex ligands such as hormones and neurotransmitters [253].

### 6.3 Summary

Reconstituting protein‐based signal‐transducing receptors in synthetic cells remains challenging, although minimal prokaryotic systems have proven more tractable [249]. DNA‐based signalling units offer a functional albeit much simpler alternative. Advances, such as control over membrane‐DNA distribution [73, 257] and logic‐gate DNA architectures for multi‐input control [254], demonstrate growing potential to introduce greater complexity. Nevertheless, proteins will likely remain superior in achieving hierarchical and orthogonal control, and integration with up‐ and downstream signalling mechanisms. Following successful protein receptor reconstitution, a promising direction may be to instead harness DNA nanotechnology to extend capabilities for synthetic cell applications. As demonstrated using living cells, DNA can spatiotemporally regulate receptor activation [63, 64, 258, 259] and reprogram specificity to enable responses to new ligands [65, 260] or mechanical forces [261]. It is also important to note that, in living cells, signal transduction is typically coupled to signal amplification and the initiation of downstream signalling cascades. To date, most studies have achieved only the initial steps: ligand binding and activation of a synthetic transmembrane receptor. Full reconstitution of signal transduction requires the co‐encapsulation and coordinated actuation of multiple downstream signalling components.

## 7 Summary and Outlook

Diverse protein and DNA membrane‐anchoring strategies have established a strong foundation for engineering signalling mechanisms in synthetic cells. Approaches based on both biomolecules have enabled key modes of communication observed in living systems, including cell–cell tethering [91, 100, 104, 134], vesicle generation [71, 157] and fusion [104, 125, 128, 134], direct transmembrane transmission [9, 214, 222], and signal transduction [249, 250, 256]. Together, these examples demonstrate that membrane‐associated signalling can be systematically reconstructed in bottom‐up synthetic systems, providing increasing control over how synthetic cells sense, communicate, and respond to their environment.

Across these signalling modalities, proteins and nucleic acids offer complementary advantages. Membrane‐associated proteins provide access to native mechanisms for membrane deformation [150, 155], transport [9, 214], and signal propagation [249]. Nucleic acid‐based systems, although initially developed by drawing inspiration from natural protein‐mediated signalling [67, 165, 222, 252, 256], now enable highly programmable, modular, and spatiotemporally controlled signalling behaviours [97, 171, 256] that are difficult to realise using proteins alone. As a result, hybrid protein‐nucleic acid architectures are emerging as a promising strategy to combine the native biological function of proteins with the programmable control afforded by nucleic acids [62, 63, 240, 243, 258, 259, 260], which is vital for real‐world application.

Despite this progress, several challenges remain before synthetic cell signalling systems can achieve the complexity and robustness of their biological counterparts. First, the reconstitution of membrane‐associated proteins into synthetic lipid bilayers remains technically demanding. Many signalling proteins require specific lipid compositions, precise membrane orientations, or accessory factors to function correctly [27, 29, 39, 40, 41, 42], and these requirements are difficult to satisfy reproducibly in bottom‐up systems. Second, while nucleic acid‐based approaches offer programmability, this typically comes at the cost of functional complexity, and closing this gap remains an open problem. Third, as the number of signalling components within a single synthetic cell increases, maintaining orthogonality between pathways becomes non‐trivial. Unintended interactions between membrane‐anchored proteins, nucleic acid networks, and encapsulated machinery are likely to undermine signal fidelity and limit the complexity of behaviour that can be reliably encoded. Fourth, most demonstrations to date remain at the level of proof‐of‐concept, establishing that a given signalling mode is achievable rather than that it is tuneable, robust, or coupled to a meaningful downstream response. Bridging the gap between signal transduction and functional cellular behaviour is a key outstanding challenge.

Looking forward, several active areas of development are expected to directly address the challenges outlined above. These include improving the efficiency and reproducibility of membrane protein reconstitution, expanding the functional complexity of nucleic acid‐based systems, and developing strategies to maintain signal orthogonality in increasingly crowded synthetic cells. De novo protein design offers a promising route to several of these reconstitution challenges [172, 262]. Computationally designed proteins could reduce reliance on accessory factors, improve compatibility with defined lipid compositions [23], and enable the creation of novel effector proteins for signal transmission, membrane fusion, and interaction with nucleic acid components [263]. Nucleic acid nanotechnology [61, 73, 257] is likewise expected to progress on multiple fronts, with increasingly complex logic‐based architectures [250, 254, 255], expanding the functional repertoire of DNA‐ and RNA‐based systems, and improvements in constructional simplicity [238], lowering the barrier to building membrane‐active assemblies. The combination of RNA‐ and DNA‐based approaches also offers a compelling route towards transcription‐coupled dynamic nanotechnology [79], providing an additional layer of temporal control over DNA‐based signalling systems. The development of up‐ and downstream signalling cascades will be equally essential for moving beyond proof‐of‐concept demonstrations. In particular, optimising intracellular enzymatic cascades to produce robust and tuneable biological outputs beyond fluorescence [196], remains challenging. Achieving systems analogous to SynNotch‐based platforms in top‐down synthetic biology [264], as recently approached in synthetic cells [5], and linking these to modular sensing and control units [21], will ultimately enable the integration of multiple orthogonal pathways within a single synthetic cell.

Beyond these advances, there are a plethora of non‐biomolecule signalling mechanisms that are being continually developed, including lipophilic ligands that induce vesicle fusion [265], synthetic amphiphilic nanopores that mediate cargo transport [19], and artificial signal transducers that convert extracellular enzymatic inputs into intracellular protein refolding responses [20]. Integrating these non‐biomolecular signalling mechanisms, with protein and nucleic acids, into synthetic cells represents an exciting direction. As the field matures, membrane‐associated signalling is likely to play an increasingly central role in enabling more autonomous, adaptive, and coordinated behaviours in synthetic cells for diverse applications.

## Funding

This study was supported by Biotechnology and Biological Sciences Research Council (BB/T008709/1, BB/W011468/1), Engineering and Physical Sciences Research Council (EP/S022856/1, EP/Y032675/1) and Royal Society (URF\R\231007).

## Conflicts of Interest

The authors declare no conflicts of interest.
